# A Prognostic Nomogram for T3N0 Rectal Cancer After Total Mesorectal Excision to Help Select Patients for Adjuvant Therapy

**DOI:** 10.3389/fonc.2021.698866

**Published:** 2021-11-25

**Authors:** Chao Zhang, Shutao Zhao, Xudong Wang

**Affiliations:** Department of Gastrointestinal Nutrition and Hernia Surgery, The Second Hospital of Jilin University, Changchun, China

**Keywords:** T3N0 rectal cancer, nomogram, prognosis, adjuvant therapy, TME

## Abstract

**Background:**

The recurrence rate of T3N0 rectal cancer after total mesorectal excision (TME) is relatively low, meaning that not all patients need adjuvant therapy (AT) (radiotherapy, chemotherapy, or chemoradiotherapy).

**Methods:**

Patients diagnosed with pT3N0M0 rectal cancer after TME were analyzed using the SEER database, of which 4367 did not receive AT and 2794 received AT. Propensity score matching was used to balance the two groups in terms of confounding factors. Cox proportional hazards regression analysis was used to screen independent prognostic factors, which were then used to establish a nomogram. The patients were then divided into three groups with X-tile software according to their risk scores. We enrolled 334 patients as external validation.

**Results:**

The C-index of the model was 0.725 (95% confidence interval: 0.694–0.756). We divided the patients into three different risk layers based on the nomogram prediction scores, and found that AT did not improve the prognosis of low- and moderate-risk patients, while high-risk patients benefited from AT. External validation data also support the above conclusions.

**Conclusion:**

This study developed a nomogram that effectively and comprehensively evaluates the prognosis of T3N0 rectal cancer patients after TME. After using the nomogram, we recommend AT for high-risk patients, but not for low- and moderate-risk patients.

## Background

Colorectal cancer is the third most common cancer and the second leading cause of cancer-related deaths worldwide, among which 2/3 of cases are colon cancer and 1/3 are rectal cancer (1, 2). Current guidelines recommend neoadjuvant chemoradiotherapy (NCRT) combined with total mesorectal excision (TME) and adjuvant therapy (AT) for locally advanced rectal cancer (RC); however, the treatment for patients with early-stage RC (T3NO) is controversial.

Although NCRT can bring better survival prognosis to RC patients, it also increases the incidence of late adverse events and postoperative complications (3, 4). Willem et al. (4) found that while NCRT reduced the local recurrence rate for resectable RC, it had no effect on overall survival (OS). Frasson et al. (5) found that T3NO RC patients did not benefit from NCRT. The local recurrence rate of T3NO RC patients is only approximately 10%. Therefore, it is now considered potentially more suitable to provide direct surgery combined with AT for patients with such low recurrence risk, thereby avoiding the side effects of overtreatment (6–10). The 5-year OS of T3N0 patients after surgery is 74–84%, and such a high survival rate means that not all patients (especially those who underwent complete radical resection) will benefit from AT. T3N0 patients’ local recurrence rate is only 3.6% after TME (9, 11). Due to the lack of randomized controlled trials (RCTs) of AT in T3N0 patients, this study focused on the clinical effect of AT in T3N0 patients at high-risk of recurrence after TME without NCRT.

This study analyzed clinicopathological factors from the SEER database and evaluated the prognosis of patients with T3N0 RC. Furthermore, the patients were divided into low-, moderate-, and high-risk groups according to a novel nomogram score to select the population that could most benefit from AT.

## Method

### Patient Cohort

SEER*Stat (version 8.3.6) software was used to search 7161 patients with pT3N0M0 RC diagnosed from 2004 to 2016. The inclusion criteria were (1): pathologically diagnosed RC (ICD-O-3: C19.9, C20.9) (2); complete follow-up and survival data (3); no NCRT (4); underwent TME; and (5) primary RC. Finally, the patients were divided into two groups according to whether they received AT: the non-AT group (*n*=4367) and the AT group (*n*=2794). The included clinicopathological variables were: age, sex, race, marital status, tumor grade, size, primary site, histology, lymph nodes retrieved, carcinoembryonic antigen (CEA) level, tumor deposits, perineural invasion, radiotherapy and chemotherapy information, and survival information. Patients were further excluded if information for the above variables was unknown.

The external validation group include 334 pT3N0M0 RC patients at our center between 2008 and 2013. The inclusion criteria and clinicopathological variables were the same as for the SEER group.

### Statistical Analysis

Associations of clinicopathological factors with the two groups were analyzed by the chi-square test. To balance potential confounding biases of the included cases, only significant clinicopathological factors according to the chi-square test were included in the propensity score matching (PSM). The non-AT group and the AT group were subjected to nearest neighbor matching according to 1:1 (12). Survival analysis was performed by the Kaplan–Meier method and the log-rank test.

### Establishing the Nomogram

First, univariate and multivariate COX analyses were performed to find correlations between the clinicopathological variables and OS in the non-AT group. Next, significant variables according to Cox multivariate analysis (*P*<0.05) were included to establish a nomogram. The effectiveness of the nomogram was tested by determining it discriminatory ability by the concordance index (C-index) (13); we also compared the C-index of the nomogram, lymph nodes retrieved, and CEA to evaluate the clinical effectiveness of the model. The calibration curve intuitively displays the consistency between the predicted survival rate and the actual survival data. Decision curve analysis (DCA) was used to evaluate the net clinical benefit as compared with lymph nodes retrieved and CEA. According to the risk score of the nomogram, all cases from the two groups were divided into three groups (high-, moderate-, and low-risk) by X-tile software (14). SPSS 24.0 (IBM, Armonk, NY, USA) and R software (version 3.5.1) were used for the statistical analyses conducted in this study, with *P*<0.05 used to denote that the difference was statistically significant.

## Results

### Patient Demographics

**Figure 1:**
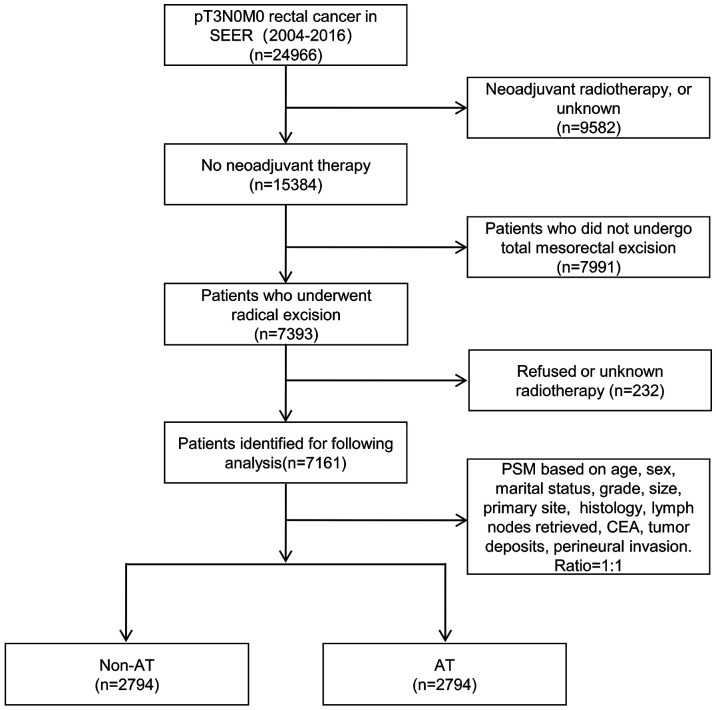
Flowchart of the selection process of included patients.

Before PSM, a total of 7161 pT3N0M0 patients who completed TME were included, including 4,367 patients without AT and 2,794 patients with AT (**Figure 1**). The median survival was 59 months (range: 0–155) and the number of deaths was 2,632 (36.8%). Chi-square analysis showed that patients with AT were significantly correlated with age, sex, marital status, grade, tumor size, primary site, histology, lymphatic invasion, CEA, tumor deposits, and perineural invasion (all *P*<0.05). After including variables related to AT for the PSM, the final patient number was 5588, including 2794 patients in the non-AT group and 2794 patients in the AT group (**Table 1**). The median survival was 64 months (range: 0–155) and there were 1,859 deaths (33.3%).

**Table 1:** Characteristics of patients.

Variable	Unmatched Cohort				Matched Cohort			P value
	Total [n(%)]	Non-AT [n(%)]	AT [n(%)]	P value	Total [n(%)]	Non-AT [n(%)]	AT [n(%)]	
Age	7161	4367	2794	<0.001	5588	2794	2794	<0.001
<65	3292	1497 (34.3)	1795 (64.2)		3291	1496 (53.5)	1795 (64.2)	
≥65	3869	2870 (65.7)	999 (35.8)		2297	1298 (46.5)	999 (35.8)	
Sex				<0.001				0.250
Male	4119	2402 (55.0)	1717 (61.5)		3392	1675 (59.9)	1717 (61.5)	
Female	3042	1965 (45.0)	1077 (38.5)		2196	1119 (40.1)	1077 (38.5)	
Race				0.875				0.279
White	5817	3552 (81.3)	2265 (81.1)		4504	2239 (80.1)	2265 (81.1)	
Black	625	384 (8.8)	241 (8.6)		523	282 (10.1)	241 (8.6)	
API	654	390 (8.9)	264 (9.4)		516	252 (9.0)	264 (9.4)	
Other	65	41 (1.0)	24 (0.9)		45	21 (0.8)	24 (0.9)	
Marital status				<0.001				0.037
Married	3947	2216 (50.7)	1731 (62.0)		3371	1640 (58.7)	1731 (62.0)	
Unmarried	1010	589 (13.5)	421 (15.1)		863	442 (15.8)	421 (15.1)	
Unknown	2202	1562 (35.8)	642 (22.9)		1354	712 (25.5)	642 (22.9)	
Grade				0.001				0.275
Well/moderately	6262	3867 (88.6)	2395 (85.7)		4817	2422 (86.7)	2395 (85.7)	
Poorly/undifferentiated	755	428 (9.8)	327 (11.7)		644	317 (11.3)	327 (11.7)	
Unknown	144	72 (1.6)	72 (2.6)		127	55 (2.0)	72 (2.6)	
Size (cm)				<0.001				0.004
<3	871	505 (11.6)	366 (13.1)		691	325 (11.6)	366 (13.1)	
≥3	5965	3702 (84.8)	2263 (81.0)		4612	2349 (84.1)	2263 (81.0)	
Unknown	325	160 (3.6)	165 (5.9)		285	120 (4.3)	165 (5.9)	
Primary site				<0.001				<0.001
Rectosigmoid junction	3884	2686 (61.5)	1198 (42.9)		2585	1387 (49.6)	1198 (42.9)	
Rectum	3277	1681 (38.5)	1596 (57.1)		3003	1407 (50.4)	1596 (57.1)	
Histology				0.034				0.303
Adenocarcinoma	6729	4117 (94.3)	2612 (93.5)		5223	2611 (93.5)	2612 (93.5)	
Mucinous adenocarcinoma	381	229 (5.2)	152 (5.4)		317	165 (5.9)	152 (5.4)	
Signet ring cell carcinoma	13	5 (0.1)	8 (0.3)		12	4 (0.1)	8 (0.3)	
Other	38	16 (0.4)	22 (0.8)		36	14 (0.5)	22 (0.8)	
Lymph nodes retrieved				0.001				0.019
< 12	2049	1183 (27.1)	866 (31.0)		1648	782 (28.0)	866 (31.0)	
≥ 12	5069	3161 (72.4)	1908 (68.3)		3907	1999 (71.5)	1908 (63.8)	
Unknown	43	23 (0.5)	20 (0.7)		33	13 (0.5)	20 (0.7)	
CEA (ng/ml)				0.001				0.170
≤5	2512	1466 (33.6)	1046 (37.4)		2045	999 (35.8)	1046 (37.4)	
>5	1625	984 (22.5)	641 (22.9)		1260	619 (22.2)	641 (22.9)	
Unknown	3024	1917 (43.9)	1107 (39.7)		2283	1176 (42.0)	1107 (39.7)	
Tumor deposits				<0.001				0.016
Negative	2836	1798 (41.2)	1038 (37.2)		2141	1103 (39.5)	1038 (37.2)	
Positive	54	23 (0.5	31 (1.1)		46	15 (0.5)	31 (1.1)	
Unknown	4271	2546 (58.3)	1725 (61.7)		3401	1676 (60.0)	1725 (61.7)	
Perineural invasion				0.027				0.300
Negative	2608	1643 (37.6)	965 (34.5)		1981	1016 (36.4)	965 (34.5)	
Positive	208	121 (2.8)	87 (3.1)		164	77 (2.8)	87 (3.1)	
Unknown	4345	2603 (59.6)	1742 (62.4)		3443	1701 (60.8)	1742 (62.4)	

AT, adjuvant therapy; API, Asian/Pacific Islander; CEA, carcinoembryonic antigen.

**Figure 2:**
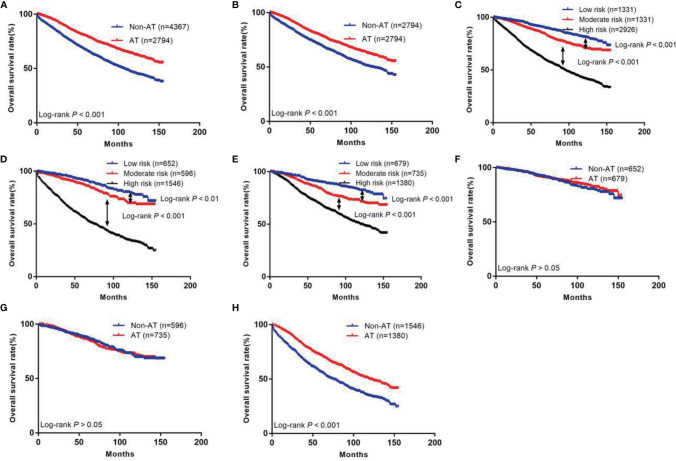
The Kaplan-Meier curves of OS for patients in our study. **(A)** All patients; **(B)** Patients after PSM; **(C)** OS in different subgroups of all patients; **(D)** OS in different subgroups of non-AT group; **(E)** OS in different subgroups of AT group; **(F)** OS for patients with or without AT in low risk group; **(G)** OS for patients with or without AT in moderate risk group; **(H)** OS for patients with or without AT in high risk group.

The prognosis of patients who received AT was better than that of the non-AT group (5-year survival rate: 79.9% *vs*. 66.8%, *P*<0.05, **Figure 2A**). After PSM, the prognosis of patients who received AT was still higher than that of the non-AT group (5-year survival rate: 79.9% *vs*. 71.0%, *P*<0.05, **Figure 2B**).

### Nomogram Construction

**Figure 3:**
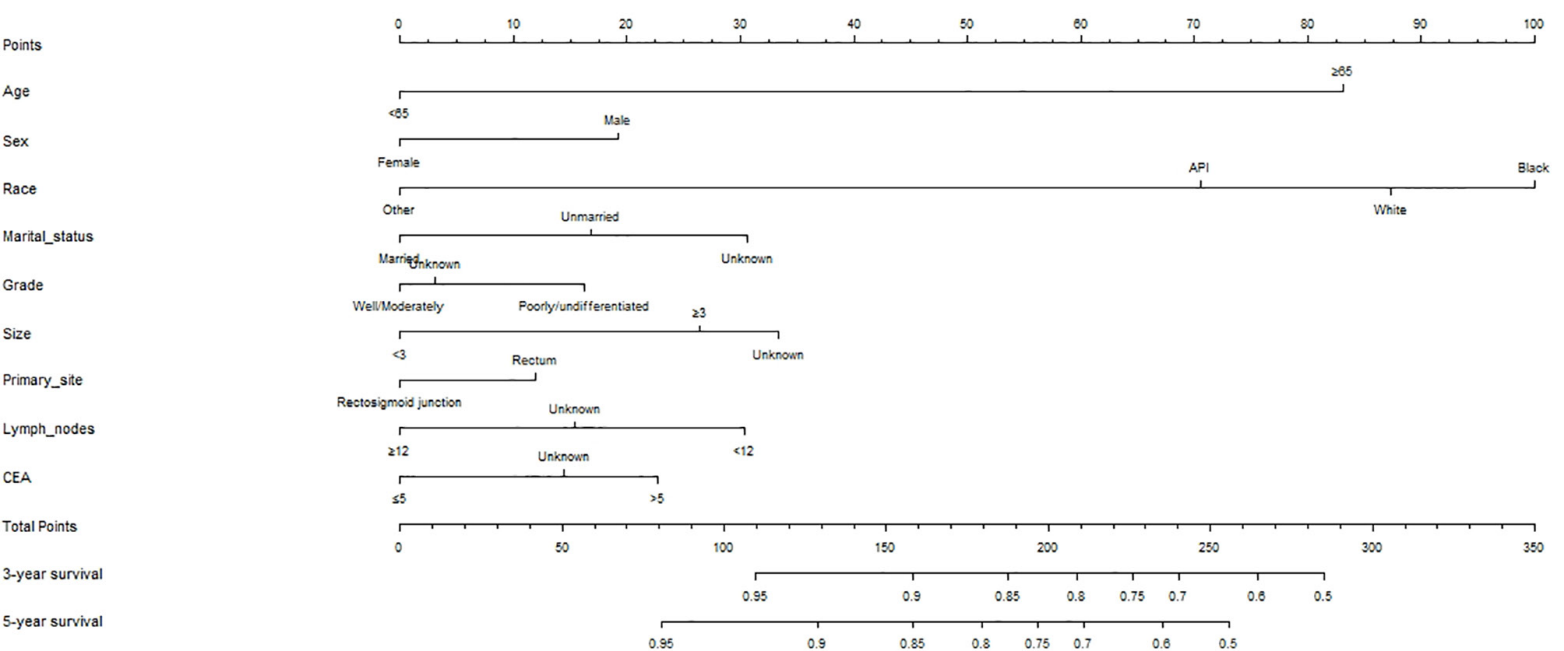
Oncologic nomogram for T3N0 rectal cancer patients after TME.

A COX hazards ratio model for patients without AT was constructed (**Table 2**), and univariate analysis showed that age, sex, race, marital status, tumor grade, size, primary site, histology, lymph nodes retrieved, CEA, tumor deposits, and perineural invasion were correlated with OS (all *P*<0.05). Next, these variables were included in the multivariate analysis, which showed that age, sex, race, marital status, tumor grade, size, primary site, lymph nodes retrieved, and CEA were independent prognostic factors (*P*<0.05). Based on these results, a nomogram was constructed to predict the 3- and 5-year survival rates of T3N0 RC after TME (**Figure 3**).

**Table 2:** The univariate and multivariate analyses of factors associated with overall survival.

Variable	univariate Cox regression		multivariate Cox regression	
	HR (95% CI)	P-value	HR (95% CI)	P-value
Age				
<65	1		1	
≥65	3.886 (3.398-4.444)	<0.001	3.421 (2.967-3.944)	<0.001
Sex				
Male	1		1	
Female	0.861 (0.760-0.974)	0.018	0.752 (0.661-0.856)	<0.001
Race				
White	1		1	
Black	1.091 (0.901-1.321)	0.371	1.205 (0.991-1.466)	0.062
API	0.653 (0.510-0.837)	0.024	0.780 (0.607-1.001)	0.051
Other	0.135 (0.019-0.956)	0.045	0.259 (0.036-1.841)	0.177
Marital status				
Married	1		1	
Unmarried	1.073 (0.897-1.284)	0.441	1.284 (1.067-1.544)	0.008
Unknown	1.680 (1.471-1.919)	<0.001	1.571 (1.366-1.806)	<0.001
Grade				
Well/moderately	1		1	
Poorly/undifferentiated	1.402 (1.183-1.662)	<0.001	1.270 (1.071-1.506)	0.006
Unknown	1.317 (0.885-1.961)	0.174	1.045 (0.700-1.561)	0.829
Size (cm)				
<3	1		1	
≥3	1.394 (1.136-1.711)	0.001	1.477 (1.198-1.819)	<0.001
Unknown	1.835 (1.334-2.524)	<0.001	1.633 (1.182-2.257)	0.003
Primary site				
Rectosigmoid junction	1		1	
Rectum	1.790 (1.582-2.025)	<0.001	1.193 (1.049-1.358)	0.007
Histology				
Adenocarcinoma	1			
Mucinous adenocarcinoma	1.400 (1.116-1.756)	0.004		
Signet ring cell carcinoma	0.712 (0.100-5.058)	0.734		
Other	2.005 (0.953-4.217)	0.067		
Lymph nodes retrieved				
< 12	1		1	
≥12	0.524 (0.464-0.591)	<0.001	0.639 (0.564-0.724)	<0.001
Unknown	0.826 (0.392-1.743)	0.616	0.800 (0.376-1.705)	0.564
CEA (ng/ml)				
≤5	1		1	
>5	1.486 (1.266-1.745)	<0.001	1.397 (1.189-1.642)	<0.001
Unknown	1.264 (1.098-1.456)	0.001	1.237 (1.073-1.426)	0.003
Tumor deposits				
Negative	1			
Positive	3.241 (1.601-6.561)	0.001		
Unknown	1.415 (1.213-1.651)	<0.001		
Perineural invasion				
Negative	1			
Positive	1.503 (0.969-2.333)	0.069		
Unknown	1.391 (1.187-1.630)	<0.001		

API, Asian/Pacific Islander; CEA, carcinoembryonic antigen.

### Testing the Effectiveness of the Nomogram

**Figure 4:**
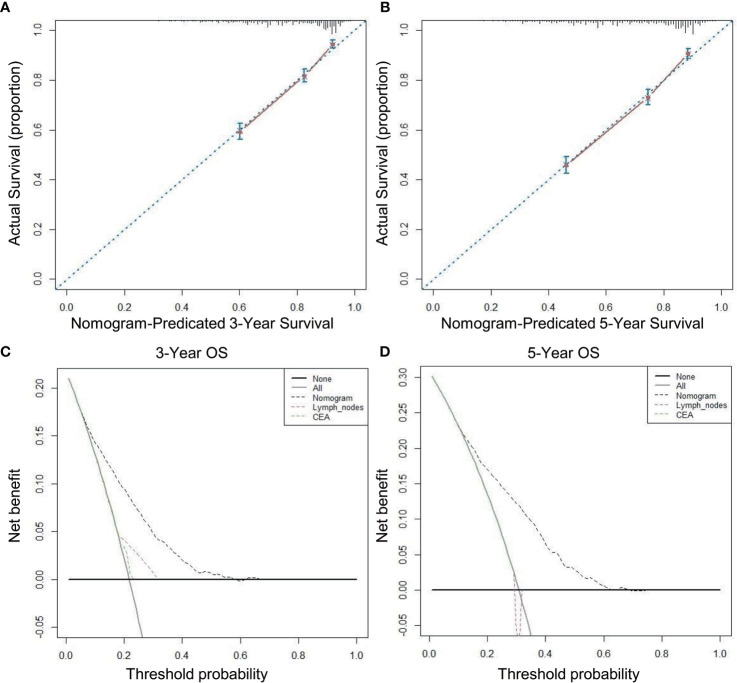
Calibration curves and decision curve for OS prediction: **(A)** 3-year OS calibration curve in our cohort; **(B)** 5-year OS calibration curve in our cohort; **(C)** Nomogram were compared to the lymph nodes retrieved and CEA in terms of 3-year OS in our decision curve analysis; **(D)** Nomogram were compared to the lymph nodes retrieved and CEA in terms of 5-year OS in our decision curve analysis.

A nomogram incorporating the above nine risk factors was constructed to judge the prognosis T3N0 RC and had a C-index of 0.725 [95% confidence interval (CI): 0.694–0.756], which is significantly higher than the C-index of prognosis judged by lymph nodes retrieved and CEA [0.581 (95% CI: 0.550–0.612) and 0.547 (95% CI: 0.514–0.580), respectively]. The calibration curve of the 3- and 5-year OS nomogram showed that the predicted survival probability was consistent with the actual survival probability. The net benefits of the nomogram for different decision thresholds were higher than those of the lymph nodes retrieved and CEA system (**Figure 4**).

### Overall Patient Risk Stratification System

**Figure 5:**
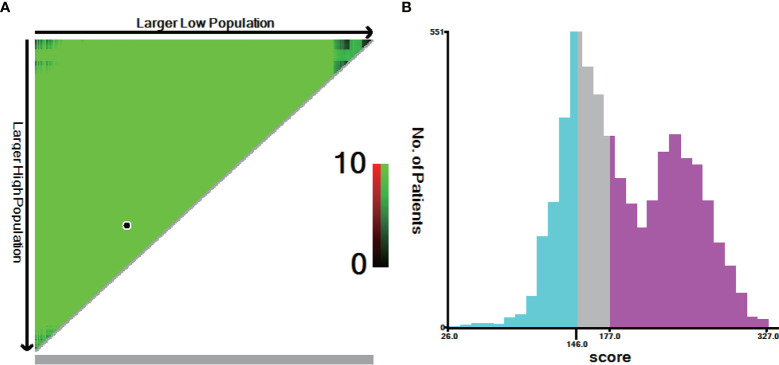
X-tile analysis for risk stratification: **(A)** The optimal cut-off value; **(B)** Numbers of patients in low, moderate and high risk subgroups.

Next, we calculated risk scores for each patient in the two groups with the nomogram (**Table 3**) and used X-tile software to take two cut-off values that divided the patients into three risk groups (**Figure 5**), a low-risk group (score ≤146, *n*=1331), a moderate-risk group (score 147–177, *n*=1331), and a high risk-group (score ≥178, *n*=2926). Five-year survival rates for the low-, moderate-, and high-risk groups were 91.2%, 86.6%, and 63.8%, respectively, which were statistically significant differences (*P*<0.001, **Figure 2C**).

**Table 3:** Point assignment of each component and prognostic score for T3N0 rectal cancer.

Group	Score	Estimated 3-y OS (%)	Estimated 5-y OS (%)
Age			
<65	0		
≥65	83		
Sex			
Male	19		
Female	0		
Race			
White	87		
Black	100		
API	71		
Other	0		
Marital status			
Married	0		
Unmarried	17		
Unknown	31		
Grade			
Well/moderately	0		
Poorly/undifferentiated	16		
Unknown	3		
Size (cm)			
<3	0		
≥3	26		
Unknown	33		
Primary site			
Rectosigmoid junction	0		
Rectum	12		
Lymph nodes retrieved			
< 12	30		
≥12	0		
Unknown	15		
CEA (ng/ml)			
≤5	0		
>5	23		
Unknown	14		
Total score			
	110	95	
	158	90	
	187	85	
	209	80	
	226	75	
	240	70	
	265	60	
	285	50	
	81		95
	129		90
	158		85
	180		80
	197		75
	211		70
	235		60
	256		50

API, Asian/Pacific Islander; CEA, carcinoembryonic antigen.

We also divided the non-AT group into three groups using the current scoring system, a low-risk group (*n*=652), moderate-risk group (*n*=596), and high-risk group (*n*=1546). Five-year survival rates of these groups were 91.8%, 87.5%, and 56.4%, respectively, which were statistically significant differences (*P*<0.01, **Figure 2D**). In the AT group, the 5-year survival rates of the low- (*n*=679), moderate- (*n*=735), and high-risk (*n*=1380) groups were 90.7%, 85.9%, and 71.8%, respectively, which were statistically significant differences (*P*<0.01, **Figure 2E**).

### Evaluating the Efficiency of AT for Patients in Different Groups

We further investigated the benefit of AT in patients with different risk stratification (**Table 4**). The results showed that patients in the low-risk group did not benefit from AT (hazard ratio [HR]: 0.89, 95% CI: 0.65–1.21, *P*>0.05, **Figure 2F**). Patients in the moderate-risk group also did not benefit from AT (HR: 1.04, 95% CI: 0.81–1.32, *P*>0.05, **Figure 2G**). In contrast, patients in the high-risk group benefited from AT (HR: 0.61, 95% CI: 0.54–0.67, *P*<0.001, **Figure 2H**).

**Table 4:** Risk stratification in non-AT and AT group.

Survival status	Non-AT Group			P value	AT Group			P value
	Low risk[n (%)]	Moderate risk [n (%)]	High risk [n (%)]		Low risk [n (%)]	Moderate risk [n (%)]	High risk [n (%)]	
Live	569 (87.3)	481 (80.7)	673 (43.5)	<0.001	595 (87.6)	580 (78.9)	831 (60.2)	<0.001
Death	83 (12.7)	115 (19.3)	873 (56.5)		84 (12.4)	155 (21.1)	549 (39.8)	

AT, adjuvant therapy.

### Evaluating the Efficiency of AT for Patients in the External Validation Group

The external validation group included 216 patients without AT and 118 patients with AT. The median survival was 83 months (range: 0–396) and the number of deaths was 192 (57.5%). The prognosis of patients who received AT was better than that of the non-AT group (5-year survival: 61.6% *vs*. 75.3%; *P*<0.001) (**Figure S1A**). According to the above scoring system, the external validation group were also divided into low, moderate, and high-risk groups. Five-year survival rates of all patients for the low-, moderate-, and high-risk groups were 92.1%, 87.4%, and 55.9%, respectively (**Figure S1B**). Five-year survival rates of non-AT group for the low-, moderate-, and high-risk groups were 83.3%, 86.5%, and 52.6%, respectively (**Figure S1C**). Five-year survival rates of AT group for the low-, moderate-, and high-risk groups were 100.0%, 88.8%, and 63.2%, respectively (**Figure S1D**).

The results showed that external validation group in the low-risk group did not benefit from AT (hazard ratio [HR]: 0.45, 95% CI: 0.05–4.33, *P*>0.05, **Figure S1E**). Patients in the moderate-risk group also did not benefit from AT (HR: 0.47, 95% CI: 0.19–1.16, *P*>0.05, **Figure S1F**). In contrast, patients in the high-risk group benefited from AT (HR: 0.63, 95% CI: 0.46–0.88, *P*=0.01, **Figure S1G**).

## Discussion

The postoperative recurrence rate of RC is as high as 40%, and the 5-year survival rate is not greater than 50% (15). TME reduces the local recurrence rate to less than 10% and increases the cancer-free survival rate to more than 70% (16, 17). Although NCRT significantly reduces the local recurrence rate to less than 7%, patients’ 5-year distant metastasis rate still exceeds 20%. The adverse reactions of NCRT may lead to a decline in quality of life and a financial burden, and delay follow-up treatment, which may lead to shorter life expectancy (18–21). For T3N0 patients with relatively low recurrence rates, the application of NCRT is controversial. A German study showed that there was no significant difference in 10-year OS (59.6% *vs*. 59.9%, *P*=0.85) between the NCRT group and the adjuvant radiotherapy group, and there was no difference in the distant metastasis rate between the two groups (29.8% *vs*. 29.6%, *P*=0.9) (22). Thus, NCRT may not be the best treatment; direct TME surgery can obtain a good prognosis and a lower postoperative recurrence rate, and TME plus AT may be an ideal treatment for patients with a higher risk of recurrence. Our study used a large sample database to screen factors that were associated with the prognosis of T3N0 patients, and then developed a nomogram to evaluate the risk score of patients and guide their AT accurately and individually.

There is still no unified view of the prognosis of young RC patients. Some studies have shown that young patients have histopathological features such as late onset, aggressive disease, and worse prognosis than older patients (23–28). But there is also a view that older patients have poor prognosis (29–31), which suggests that age is a controversial prognostic factor in RC. Our study found that patients ≥65-years old had a poor prognosis (HR: 3.42, 95% CI: 2.97–3.94, *P*<0.001). One possible reason is that older patients are less sensitive to sensation, which makes the clinical manifestations of RC in the elderly more atypical and easier to ignore, resulting in later staged disease in many older patients. In addition, the proportion of the elderly who have received radical surgery is low, due to the poor tolerance to surgery and a large number of comorbidities, leading to a higher incidence and mortality of perioperative diseases in the elderly and a poor prognosis.

Studies have shown that colorectal cancer incidence is higher in men than in women, which may be associated with estrogen levels. Young and middle-aged women with higher estrogen levels have a decreased risk of colorectal cancer risk, and the cumulative protection of higher estrogen levels can be extended up to 20 to 25 years after menopause (32–34). Our study was consistent with the literature in that female patients had a better prognosis (HR: 0.75, 95% CI: 0.66–0.86, *P*<0.001) (35).

Pulte et al. (36) found that blacks and Indians had worse outcomes than whites, which is consistent with our results. Our study found that the prognosis of unmarried patients was worse than that of married patients, consistent with previous studies (37–39), which may be related to the lower proportion of unmarried patients participating in RC screening, lower enthusiasm for treatment, and lower proportion of patients receiving surgery and AT.

Currently, serum CEA is the most important tumor marker applied in clinical colorectal cancer management. Serum CEA levels can predict the prognosis and recurrence of colorectal cancer, and the later the disease stage is, the higher serum CEA levels are, and increased CEA is correlated with poor tumor differentiation (40–42). Our study also found that CEA (HR: 1.40, 95% CI: 1.19–1.64, *P*<0.001) and poor tumor differentiation (HR: 1.27, 95% CI: 1.07–1.51, *P*=0.006) are poor prognostic factors for T3N0 RC.

Tumor size is related to the time of tumor existence, invasion, and distant metastasis, and therefore, also to poor prognosis (43). Our study found that the prognosis of tumors ≥3 cm was worse (HR: 1.48, 95% CI: 1.20–1.82, *P*<0.001). As the boundary between the colon and rectum, rectosigmoid junction cancer may be different from RC and colon cancer in terms of pathogenesis, treatment, and prognosis. It is generally believed that the prognosis of diploid DNA tumors is better than that of aneuploid tumors. Diploid status was more common in proximal colorectal cancer than in distal colorectal cancer. The benefit of 5-FU treatment is greater for proximal colorectal cancer, but less for distal colorectal cancer. Therefore, from proximal colorectal cancer to distal colorectal cancer to RC, the prognosis of patients is gradually worse (44). Our study also confirmed that the prognosis of RC was worse than that of rectosigmoid junction cancer (HR: 1.19, 95% CI: 1.05–1.36, *P*=0.007).

The number of lymph nodes retrieved after surgery is closely related to the postoperative pathological stage of RC patients. Retrieving few lymph nodes may be related to an insufficient degree of lymph node dissection during surgery, and even lead to lymph nodes that are positive in the surgical area are not dissected, which affects prognosis. It is suggested in the guidelines that at least 12 lymph nodes should be detected to ensure that there is no bias in staging (45, 46). There is already evidence that in patients with lymph node-negative colorectal cancer, a higher number of lymph nodes retrieved is associated with prognosis (47). Many studies have found that in stage II/III colorectal cancer, the prognosis of patients with <12 lymph nodes retrieved is worse than that of patients with >12 lymph nodes retrieved (48–51). Our study also found that the prognosis of patients with >12 lymph nodes retrieved was relatively better (HR: 0.64, 95% CI: 0.56–0.72, *P*<0.001). New lymph node staging indicators such as metastatic lymph nodes ratio, log odds of positive lymph nodes, negative lymph node count, and lymph node micrometastasis may predict prognosis more accurately (52–56).

Our nomogram of multiple prognostic factors was established through large sample data and more comprehensively incorporates factors that affect the prognosis of T3N0 than the number of lymph nodes retrieved [C-index: 0.581 (95% CI: 0.550–0.612)], and CEA [C-index 0.547 (95% CI: 0.514–0.580)], and our nomogram [C-index: 0.725 (95% CI: 0.694–0.756)] better predicts the 3- and 5-year survival rates of T3N0 RC. We applied DCA to further confirm that the nomogram was superior to the number of lymph nodes retrieved and CEA in predicting the OS of T3N0 RC patients after TME.

Whether T3N0 RC can benefit from AT is controversial, and so far, no RCT has studied whether T3N0 RC can benefit from AT. Paula et al. (10) found that postoperative adjuvant chemotherapy had no survival benefit compared with patients without chemotherapy (HR: 0.88, 95% CI: 0.52–1.56, *P*=0.66). Kim et al. (57) found that the 5-year OS of patients receiving postoperative adjuvant chemotherapy was lower than that of patients without chemotherapy (79.3 *vs*. 83.0, *P*=0.92). However, Quinn et al. (9) found that postoperative chemotherapy (HR: 0.74, 95% CI: 0.62–0.89, *P*=0.001) and chemoradiotherapy (HR: 0.57, 95% CI: 0.50–0.65, *P*<0.001) improved the prognosis of patients compared with surgery alone. Our findings suggest that AT improved patient survival both before and after PSM. Because AT is often used in patients with poor prognostic factors, they benefit more from AT, resulting in the overall results showing that AT improves prognosis. However, this does not mean that all patients need AT, which requires us to select those who will really benefit from AT for precise and individualized treatment. Our nomogram comprehensively analyzed factors that influence the prognosis and recurrence of T3N0 RC, and scored the impact of each risk subgroup: low, moderate, and high. Moreover, in our non-AT and AT groups, there were obvious survival differences in the low-, moderate-, and high-risk subgroups, indicating that our risk stratification was reasonable and effective. To determine which subgroups of patients benefit from AT, we found that the 5-year survival rate of low-risk patients receiving AT was lower than that of patients without AT (90.7% *vs*. 91.7%, *P*>0.05), so we do not recommend AT for low-risk patients. The 5-year survival rate of patients with moderate risk who received AT was lower than that of patients without AT (85.9% *vs*. 87.5%, *P*>0.05). We also do not recommend AT for such patients because the harm of AT for low and moderate risk patients exceeds the benefit. The 5-year survival rate of high-risk patients who received AT was higher than that of patients without AT (71.8% *vs*. 56.4%, *P*<0.001), we suggest that high-risk patients receive AT. Our external validation data also showed that low and moderate -risk patients did not benefit from AT (*P*>0.05), while high-risk patients did (*P*<0.05).

This study has several limitations. This was a retrospective study, and some patients fail to be included in this study due to missing data, which may cause bias. Currently, there is no large-scale RCT study on whether T3N0 RC benefits from AT, and there is no prognostic survival nomogram that incorporates the above clinical pathological factors. The most important thing is that we use this nomogram to stratify the risk of patients, which is of great significance for individualized guidance of clinical AT, as was the goal of this work.

## Conclusion

Age, sex, race, marital status, tumor grade, size, primary site, lymph nodes retrieved, and CEA are independent prognostic factors for T3N0 RC patients after TME. Through our innovative risk score stratification, we recommend high-risk patients receive AT, while AT is not recommended for low- and moderate-risk patients.

## Data Availability Statement

The original contributions presented in the study are included in the article/**Supplementary Material**. Further inquiries can be directed to the corresponding author.

## Ethics Statement

Ethical review and approval was not required for the study on human participants in accordance with the local legislation and institutional requirements. Written informed consent for participation was not required for this study in accordance with the national legislation and the institutional requirements. The ethics committee waived the requirement of written informed consent for participation.

## Author Contributions

XW designed the research. SZ took part in designing the research. SZ collected the data, analyzed the date. CZ analyzed the date and wrote the manuscript. All authors contributed to the article and approved the submitted version.

## Funding

This work was supported by Department of Finance of Jilin Province (No 2020SCZT031).

## Conflict of Interest

The authors declare that the research was conducted in the absence of any commercial or financial relationships that could be construed as a potential conflict of interest.

## Publisher’s Note

All claims expressed in this article are solely those of the authors and do not necessarily represent those of their affiliated organizations, or those of the publisher, the editors and the reviewers. Any product that may be evaluated in this article, or claim that may be made by its manufacturer, is not guaranteed or endorsed by the publisher.

## Supplementary Material

Supplementary Figure 1 The Kaplan-Meier curves of OS for patients in external validation group. **(A)** All patients; **(B)** OS in different risk subgroups of all patients; **(C)** OS in different risk subgroups of non-AT group; **(D)** OS in different risk subgroups of AT group; **(E)** OS for patients with or without AT in low risk group; **(F)** OS for patients with or without AT in moderate risk group; **(G)** OS for patients with or without AT in high risk group.
