# Supplementary figures and images for: A Prognostic Nomogram for T3N0 Rectal Cancer After Total Mesorectal Excision to Help Select Patients for Adjuvant Therapy
Source: Front Oncol. 2021 Nov 25;11:698866. doi: 10.3389/fonc.2021.698866 (PMC8654784; doi:10.3389/fonc.2021.698866)

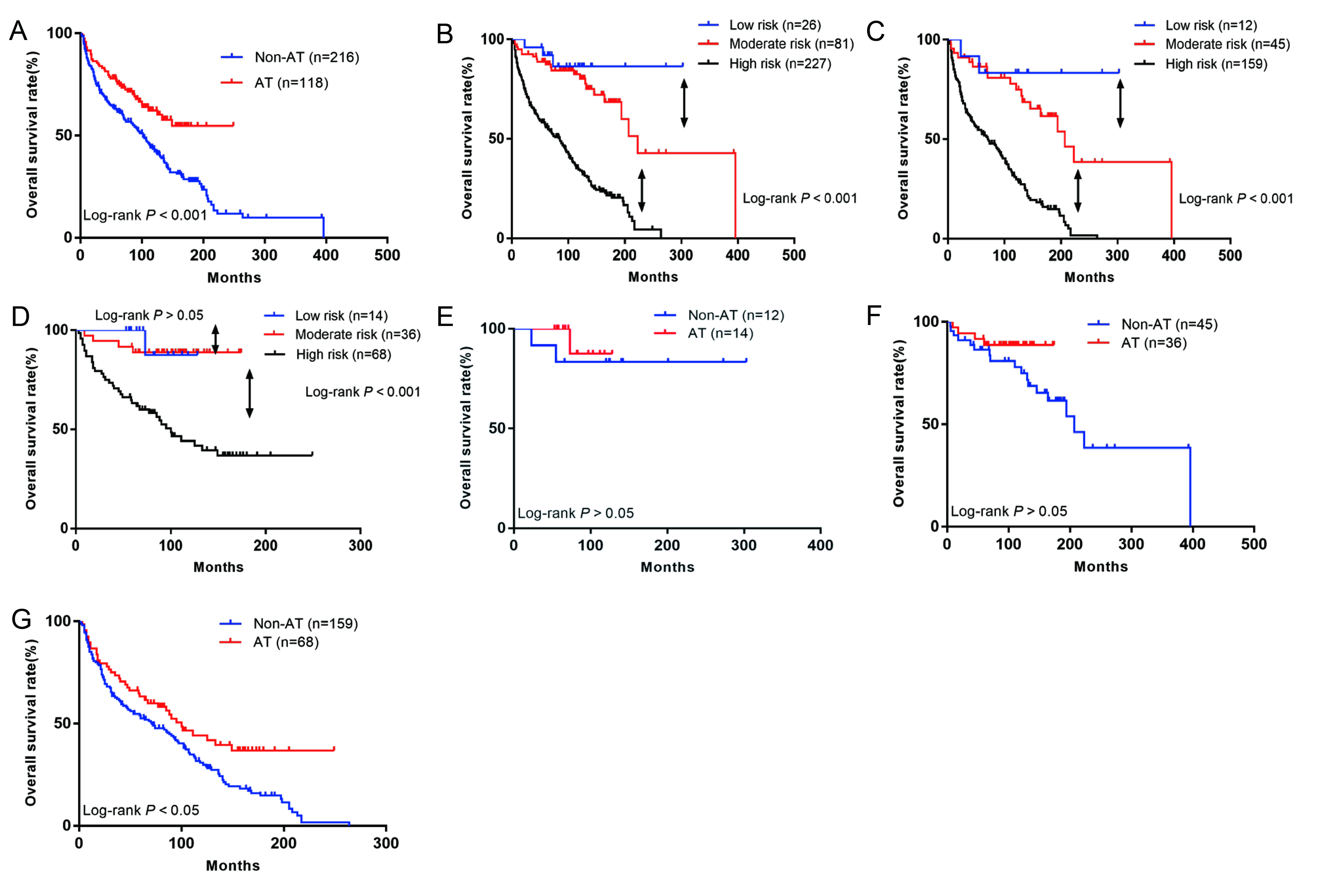

Supplement: Supplementary Figure 1 — The Kaplan-Meier curves of OS for patients in external validation group. (A) All patients; (B) OS in different risk subgroups of all patients; (C) OS in different risk subgroups of non-AT group; (D) OS in different risk subgroups of AT group; (E) OS for patients with or without AT in low risk group; (F) OS for patients with or without AT in moderate risk group; (G) OS for patients with or without AT in high risk group. [file Image_1.tif]
